# PEPFAR Transitions to Country Ownership: Review of Past Donor Transitions and Application of Lessons Learned to the Eastern Caribbean

**DOI:** 10.9745/GHSP-D-14-00227

**Published:** 2015-06-12

**Authors:** Abigail Vogus, Kylie Graff

**Affiliations:** ^a^​ Abt Associates, Inc, Bethesda, MD, USA

## Abstract

The US President’s Emergency Plan for AIDS Relief (PEPFAR) has shifted from an emergency response to a sustainable, country-owned response. The process of transition to country ownership is already underway in the Eastern Caribbean; the Office of the US Global AIDS Coordinator (OGAC) has advised the region that PEPFAR funding is being redirected away from the Eastern Caribbean toward Caribbean countries with high disease burden to strengthen services for key populations. This article seeks to highlight and apply lessons learned from other donor transitions to support a successful transition of HIV programs in the Eastern Caribbean. Based on a rapid review of both peer-reviewed and gray literature on donor transitions to country ownership in family planning, HIV, and other areas, we identified 48 resources that addressed key steps in the transition process and determinants of readiness for transition. Analysis of the existing literature revealed 6 steps that could help ensure successful transition, including developing a clear roadmap articulated through high-level diplomacy; investing in extensive stakeholder engagement; and supporting monitoring and evaluation during and after the transition to adjust course as needed. Nine specific areas to assess a country’s readiness for transition include: leadership and management capacity, political and economic factors, the policy environment, identification of alternative funding sources, integration of HIV programs into the wider health system, the institutionalization of processes, the strength of procurement and supply chain management, identification of staffing and training needs, and engagement of civil society and the private sector. In the Caribbean, key areas requiring strengthening to ensure countries in the region can maintain the gains made under PEPFAR include further engaging civil society and the private sector, building the capacity of NGOs to take on essential program functions, and maintaining donor support for targeted capacity building and long-term monitoring and evaluation efforts.

## Abstract (precis)

Six key steps for effective transition: (1) develop a roadmap; (2) involve stakeholders; (3) communicate the plan; (4) support midterm evaluations; (5) strengthen financial, technical, and management capacity; and (6) support ongoing M&E. The Eastern Caribbean will need to identify HIV champions; strengthen leadership and management; improve policies to protect key populations; engage the private sector and civil society more; integrate HIV programs into primary care; improve supply chain capacity; and address health worker shortages.

## INTRODUCTION

The US President’s Emergency Plan for AIDS Relief (PEPFAR) has helped introduce lifesaving treatment to 7.7 million people living with HIV (PLHIV) worldwide.^1^ Since its inception, the program has shifted from an emergency response against a growing epidemic (PEPFAR I, from 2003–2007) to a more targeted, sustainable approach with greater country ownership (PEPFAR II, from 2008–2012).^2^ PEPFAR 3.0 (2013–2019) seeks to maximize the impact of investments by targeting evidence-based interventions for key geographic areas and populations with the highest incidence rates.^3^ Its goal is to accelerate progress toward epidemic control and sustain achievements and gains.^4^

The Sustainability Agenda, a key dimension of PEPFAR 3.0’s new business model, acknowledges that long-term epidemic control and maintenance of the progress to date will require country-owned responses.^4^–^5^ PEPFAR’s new Country Operational Plan (COP) Guidance requires countries to assess progress over time in 5 domains of sustainability: (1) availability of current data for decision making; (2) local leadership in service delivery; (3) domestic health financing and resource mobilization; (4) accountability for and transparency of results and spending; and (5) an enabling environment (appropriate policies, laws, regulations, as well as effective planning and coordination) for successful program implementation.^4^ Countries must now implement strategies that improve program sustainability and capacity to manage, lead, coordinate, and implement national HIV programs.

PEPFAR’s new business model recognizes that long-term control of the HIV epidemic will require country-owned responses.

In 2013, in preparation for regional PEPFAR planning, the US Agency for International Development (USAID) Mission in Barbados and the Eastern Caribbean requested assistance from Abt Associates in framing the transition of PEPFAR programming to country ownership in the Caribbean with an eye toward greater sustainability (Box). Specifically, we were tasked with gathering and analyzing findings from other donor transitions, including graduation from USAID family planning programs in Latin America and the Caribbean and from current PEPFAR transitions, to identify key themes and lessons learned that might be applied to the Caribbean. The Office of the US Global AIDS Coordinator (OGAC) has since published PEPFAR 3.0 and the revised COP guidance. OGAC has also advised the Caribbean of a shift in the program’s strategy, focus, and geographical footprint. Under the new strategy, funding is being redirected away from the Eastern Caribbean toward Caribbean countries with higher disease burden to strengthen services for key populations.

**BOX. Defining “Transition to Country Ownership”**

For the purposes of this review, we used the Global Health Initiative's definition of country ownership^5^:

… *the continuum of actions taken by political and institutional stakeholders in partner countries to plan, oversee, manage, deliver, and finance their health sector. These actions advance sustainable, quality health programs that are locally owned and responsive to the needs of host country nationals.*

We acknowledge that country ownership requires a comprehensive response from the public, private for-profit, private not-forprofit, and civil society communities. We define “transition to country ownership” as the conscientious shifting of donor-led health initiatives to country ownership.^5^

This article helps support these new developments by identifying themes and lessons learned from our literature review to successfully plan, develop, and implement transition strategies that can translate across all PEPFAR countries. Many of the determinants for successful transition identified in the literature closely align with those outlined in PEPFAR’s recent guidance.^4^ In this article, we place specific emphasis on applying the findings from the literature on successful transition to the Caribbean context in preparation for the shift from the PEPFAR-Caribbean Regional Partnership Framework (“Partnership Framework”) (2010–2014) toward programs with greater country ownership.

## PROFILE OF THE CARIBBEAN REGION

Comprised of a series of small island nations and mainland countries, the Caribbean has the second highest regional HIV prevalence rate in the world behind sub-Saharan Africa. Adult HIV prevalence among Partnership Framework countries, namely Antigua and Barbuda, Bahamas, Barbados, Belize, Dominica, Grenada, Jamaica, St. Kitts and Nevis, St. Lucia, St. Vincent and the Grenadines, Suriname, and Trinidad and Tobago, is approximately 1%. The epidemic is primarily concentrated among key populations, including commercial sex workers (CSWs), men who have sex with men (MSM), and prisoners.^6^ A low regional average masks much higher estimated prevalence rates among these key populations. Research shows that seroprevalence rates among CSWs are 9% in Jamaica and 21% in Suriname.^7^ Unprotected sex between men accounts for at least 10% of HIV infections in the Caribbean and as high as 30% of infections in Jamaica.^8^

The HIV epidemic in the Caribbean is concentrated primarily among commercial sex workers, men who have sex with men, and prisoners.

The Caribbean’s geopolitical, economic, and cultural context presents unique challenges when planning sustainable, country-owned responses. Although the region is comprised predominantly of middle-income countries, many of the countries rely significantly on PEPFAR funding for HIV programming. For example, recent national health accounts exercises in Dominica and St. Kitts and Nevis indicate that international donors provide 27% and 47% of HIV funding, respectively.^9^^,^^10^ Over the course of the Partnership Framework period, this funding has supported counseling and testing services for nearly 175,000 individuals and care and support services for more than 4,000 people.^11^ National human and financial resources are oftentimes directed toward chronic noncommunicable diseases, which account for almost 8% of gross domestic product in some Caribbean countries.^12^ National budgets also help address emerging health threats such as chikungunya and Ebola. Meanwhile, financial assistance from the US government (USG) to support health initiatives in the Latin American and Caribbean region declined 31% between fiscal years 2011 and 2014.^13^ The USG has since communicated its intentions to further divert HIV funding away from Eastern Caribbean countries with lower prevalence rates (letter from Larry Palmer, US Ambassador to the Eastern Caribbean and the Organization of Eastern Caribbean States, 13 Aug 2014).

Small population bases limit the number of qualified health workers available to implement HIV interventions. With the exception of Jamaica (19 per 10,000 population), English-speaking Caribbean countries meet the World Health Organization’s benchmark of 25 trained health professionals (physicians, nurses, midwives) per 10,000 population.^14^ However, most countries still claim human resource shortages, citing brain drain, particularly among experienced, midlevel health professionals.^14^ A 2007 World Bank study estimated a shortage of approximately 7,800 nurses in the English-speaking Caribbean; the shortage is expected to increase to roughly 10,700 nurses by 2025 due to the health needs of a rapidly aging population.^15^

Culturally, small population sizes with largely conservative religious influences reduce confidentiality and cultivate widespread stigma and discrimination against PLHIV. This is exacerbated by conservative public policies that outlaw transactional sex and criminalize homosexuality. The result is largely isolated key populations unwilling or unable to access essential HIV prevention, care, and support services from the public health sector. This has placed the onus of reaching key populations on an NGO sector that is often small and fragmented. Since the beginning of the Partnership Framework, PEPFAR has helped these NGOs deliver HIV prevention interventions to nearly 57,000 individuals in key populations.^11^ Many of these NGOs are now heavily reliant upon PEPFAR funding and require additional capacity-building efforts to support long-term organizational viability and continued access to services for key populations.

NGOs, with support from PEPFAR, have been instrumental in reaching key populations with HIV services.

## METHODS

We sought to answer the following question:

*What lessons can be drawn from existing literature to help ensure an effective transition of HIV programs to country ownership, and what determinants can be used to assess the Caribbean’s readiness for such a transition?*

To answer this question, we conducted a rapid review of both peer-reviewed and gray literature with expansive search terms to reflect the shifting language and changing contexts around donor transitions to country ownership. We initially focused on articles discussing the “withdrawal” of donor funding but later expanded the search terms to include “country ownership,” “graduation,” and “sustainability” to address the expanding definition of transition beyond self-financing HIV programs. For example, USAID’s transition away from funding large family planning programs in Latin America and the Caribbean is typically referred to as graduation. While the term graduation was also used in early discussions around PEPFAR transitions, there has been a steady movement in the dialogue toward country ownership, country-owned responses, and sustainability. We searched Google and PubMed for English-language publications using combinations of these terms to ensure a more comprehensive literature review (Table 1).

**TABLE 1.** Key Search Terms Used on Google and PubMed

**Donor Funding Search Terms (Using “OR” Boolean Operator)**		**Transition Search Terms (Using “OR” Boolean Operator)**		**Health Area Search Terms (Using “OR” Boolean Operator)**
Donor	AND	Withdrawal	AND	HIV
Development assistance		Graduation		Family planning
Funding		Transition		[none]
		Sustainability		
		Country ownership		

Using these search terms, we scanned existing literature, including reports, case studies, and scholarly journal articles, and reviewed abstracts and executive summaries to determine relevance to the research question. Those selected for inclusion focused on the shifting dynamic of health programming between one or more donor agencies and partner countries and addressed at least one of the following questions:

- What does or could transition to country ownership mean within the PEPFAR context?
- What are key steps in the transition to country ownership?
- What actions make for a successful transition to country ownership?

We identified 48 resources that matched these criteria (Table 2). Then we read and analyzed the full text of the selected resources to determine key steps required to implement a successful transition to country ownership and to identify potential determinants of readiness for transition. We also supplemented the findings with anecdotal evidence from our experience working in the Eastern Caribbean to assist USAID/Barbados and the Eastern Caribbean to better tailor its transition-planning process. The initial review was conducted in January 2013 and then updated in May 2014 and February 2015 to prepare for this publication.

**TABLE 2.** Publication Types and Methods of Analysis

**Resource Type**	**No. Reviewed**	**Citations**	**Method(s) of Analysis**
Reports	25	9, 10, 13, 16–18, 20, 21, 23–25, 28, 31–36, 38, 40, 44–48	Quantitative data analysis, qualitative data analysis, key informant interviews, literature reviews
Scholarly journal articles	10	22, 26, 27, 29, 30, 37, 39, 41–43	Quantitative data analysis, qualitative data analysis, key informant interviews, literature reviews
Policy documents	8	2–5, 7, 14, 15, 19	Policy directives, key informant interviews, literature reviews
Fact sheets	4	1, 6, 8, 12	Secondary quantitative analysis
Online databases	1	11	Secondary quantitative analysis
**TOTAL**	**48**		

## RESULTS

### Key Steps in Transitioning to Country Ownership

Our analysis of existing literature revealed a series of 6 key steps in planning an effective transition to country ownership: (1) develop a roadmap; (2) invest in stakeholder participation; (3) communicate the plan through high-level diplomacy; (4) support midterm evaluations; (5) provide technical assistance throughout the process; and (6) provide long-term monitoring and evaluation (M&E) support.

#### 1. Develop a Roadmap

Concise roadmaps are necessary to clearly communicate transition goals and processes. Findings from the literature revealed a lack of clarity among stakeholders about what transition to country ownership meant in practical terms. A clear strategy did not exist in the early stages of graduation from family planning programming in the 1990s; a published review of those early graduations revealed that formal strategies were essential to successful transitions, resulting in establishment of a systematic process by 2004.^16^ In South Africa, the Center for Strategic and International Studies found that lack of a written plan and clear communication about PEPFAR’s transition created substantial resentment and frustration among South African officials.^17^^,^^18^ The absence also contributed to skepticism among civil society organizations (CSOs) that the impetus of the transition was PEPFAR withdrawal amidst USG budget cuts.^18^ South Africa’s Partnership Framework made it clear that funding would decrease over time, but it did not specify the pace of the reductions.^18^ The plan also focused primarily on the transition of care and treatment but did not discuss what the transition would mean for prevention activities. This resulted in largely underfunded prevention programs and a lack of sufficient focus on high-impact interventions and strategies that were historically funded by donors.^18^ Africa’s experience highlights the need for clear guidance on *all* HIV program areas. This is a critical lesson for Caribbean roadmap development, as prevention programs are widely supported by international donors.

Roadmaps are needed to clearly communicate transition goals and processes.

One potential approach to optimize success is to develop 2 separate roadmaps. The first roadmap could be within PEPFAR to outline the basic process for transition planning. For example, PEPFAR’s “FY2014 Sustainability Planning Guidance Document: Advancing Country Ownership in PEPFAR III” (“Sustainability Planning Guidance”) outlines PEPFAR’s approach to achieving high-impact national HIV responses that maintain service levels and quality under the ownership of “government, civil society, the private sector, and other stakeholders in the partner country.”^5^ The second roadmap could be country- and/or region-specific and negotiated with country-level stakeholders. These roadmaps could lay out the shared responsibilities between USG agencies and local governments, donors, and other stakeholders, including intended funding levels.^16^ This plan could clearly outline expectations, objectives, activities, timelines, and human and financial resource commitments among all stakeholders while indicating the seriousness of donor withdrawal. A mutually agreed upon roadmap of this nature would promote transparency between donor and recipient countries while minimizing misconceptions.^16^

#### 2. Invest in Stakeholder Participation

Stakeholder participation is a vital component of any successful transition-planning process.^18^^–^^22^ A variety of stakeholders should be involved, including high-level diplomats, officials from the Ministry of Health and Ministry of Finance, CSOs, other donors, and private-sector representatives. Involvement of country stakeholders increases the likelihood that counterparts at all levels buy into the plan, understand its intentions, and accept stakeholder responsibilities.^22^ Country counterparts need to own the process of mobilizing new resources and shaping the next phase of PEPFAR-partner country relations.

Involving stakeholders in the planning process increases the chances of their buy-in but requires more time for the transition.

Intensive stakeholder participation will require a longer time frame for transition. According to Slob and Jerve, no less than 2 years is required to sufficiently involve the full set of key stakeholders in the transition-planning process.^19^ In Mexico, family planning graduation was originally planned as a 5-year process. Two additional years were ultimately added to transition full ownership of family planning programs.^26^ In South Africa, health practitioners have already cautioned that while country ownership is essential to long-term sustainability, a hasty transition could undercut access to services.^26^^,^^27^ South Africa’s experience to date has shown that the speed of PEPFAR’s withdrawal of human resources and funding has seriously disrupted treatment to an estimated 50,000 to 200,000 PLHIV.^18^

#### 3. Communicate Transition Strategies Through High-Level Diplomacy

Leaders from donor and recipient countries should be viewed as active partners with shared, consistent core messaging around why and how transition will happen.^17^ Messaging should also acknowledge challenges to successful transition. Donors, for example, should clearly communicate the method in which PEPFAR funds are approved by Congress and the uncertainties this may cause in the resource-allocation process. Early reports from the family planning graduation process found that many countries also experienced mixed messaging from donors about funding timelines. This contributed to unwise resource utilization because of misunderstanding over how long funding would be provided.^16^ Developing messaging through high-level diplomacy helps alleviate these issues and strengthens country engagement by encouraging active stakeholder participation while stressing the seriousness of proposed donor withdrawals.^17^^,^^20^^,^^21^

#### 4. Support Midterm Evaluations and Allow Flexibility

Midterm assessments provide an opportunity to validate initial assumptions underlying transition plans and to respond to emerging challenges. Early reviews of family planning graduations found that the most successful transition plans were flexible to accommodate changing needs and contexts, oftentimes identified via midterm assessments.^16^^,^^21^^,^^24^ A critical failure identified in South Africa’s transition is the lack of a system to track patients who may be lost to follow-up as services shift from NGO partners to government clinics.^18^ The lack of a tracking system means that the exact size, scope, and location of the problem is largely unknown, making it virtually impossible to make midcourse corrections. In Brazil, the family planning transition incorporated a midterm assessment, which validated the strategy and recommended additional management components for 2 states.^16^ In Mexico, a midterm assessment led to an extension of the phase-out time frame.^16^

The most successful transitions are often flexible to accommodate changing needs and contexts identified through midterm assessments.

#### 5. Provide Technical Support to Implement the Plan

According to Slob and Jerve, institutional capacity to manage donor withdrawal is a key factor in determining transition success. Initial assessments of financial and technical capacity can help tailor an appropriate roadmap for donor withdrawal and reveal which areas require additional support to fully manage HIV activities and integrate them into national health plans.^21^ An evaluation of the phase out of family planning support in Mexico revealed that donors should have attempted to institutionalize technical capacity for such key program areas as commodity procurement.^24^ Case studies in Botswana and Malawi also highlighted that assessing and addressing capacity issues facilitated more successful transitions.^21^ As was suggested for transition in South Africa, PEPFAR should consistently commit to providing capacity-building support to strengthen overall program management and successful program transition.^17^

#### 6. Provide Ongoing M&E Support

A sustainable program is one in which a country can maintain or improve priority health outcomes. The outcomes can be compromised by new health challenges, unexpected instability, or overestimation of in-country capacity after donor withdrawal. Not all countries have the financial resources and technical expertise to measure these outcomes. Population-based surveys, such as the Demographic and Health Surveys (DHS), are usually sponsored by donors. Supporting ongoing M&E will be especially important in the Caribbean where accurate seroprevalence data is scarce. Ongoing funding for research and health outcomes measurement should be incorporated into the transition roadmap to assist the country in monitoring progress, to help measure the USG’s own success in transitioning, and to contribute to global health research agendas.^20^^,^^24^ Cromer et al. note that such support will also reinforce the ongoing partnership between the USG and the partner country after direct program assistance is withdrawn.^16^^,^^25^

### Determinants of Readiness for Successful Transition to Country Ownership

In February 2015, PEPFAR released new guidance for developing country/regional operational plans, including a “Sustainability Index and Dashboard” (SID) tool to assess sustainability of country programs toward control of the HIV epidemic.^4^ The SID tool and Sustainability Planning Guidance are critical in clarifying the proposed process of achieving sustainable, country-owned programs. They more clearly articulate dimensions for successful country ownership, including political ownership and stewardship, institutional and community ownership, capabilities, and mutual accountability, including financing. Other tools such as the “Capacity Assessment Tool for Country Ownership of HIV Care and Treatment” have also offered frameworks to assess the capacity of a country or province to take on greater responsibility in the planning, organization, and management of HIV programs.^5^

This section expands on existing guidance by describing 9 key areas, based on findings from the literature, which should be evaluated when determining readiness for transition to country ownership. We also apply these principles to the Caribbean’s unique regional context to assess readiness and potential barriers to success that may require additional support. Examples focus on countries in the Organization of Eastern Caribbean States (OECS) (Antigua and Barbuda, Dominica, Grenada, St. Kitts and Nevis, St. Lucia, and St. Vincent and the Grenadines).

#### 1. Leadership and Management Capacity

Country ownership of the national HIV response requires identifying advocates who will promote the cause.^19^^,^^28^^,^^29^ Government decision makers must identify HIV programming as an essential part of health services and advocate national funding for such programming using accurate and compelling analytics. In the Caribbean, competing health priorities, stigma and discrimination, and relatively small affected populations make it especially difficult to gain the attention of decision makers. While most leaders recognize that care and treatment for HIV is important, it is politically challenging to invest significant resources, financial and otherwise, into their HIV response without first advancing chronic noncommunicable disease programs. Successful transitions will need to identify champions for the process.

A sustained, country-owned response to HIV requires champions who will be advocates for the cause.

Most research on sustainability and country ownership has highlighted the need for increased management capacity. Where PEPFAR has provided more direct provision of services, local partners may need further training in such key management areas as health planning, M&E, procurement, performance management, and financial management.^19^^–^^21^^,^^26^^,^^30^ A series of health systems and private-sector assessments in the OECS found that overall management capacity in the region is limited and few officials have planning or health financing backgrounds.^31^^–^^36^ Organizations such as the Caribbean HIV/AIDS Regional Training Network and Caribbean Health Leadership Institute are building local capacity in leadership and management. However, these organizations rely heavily on PEPFAR funding and require continued financial support to maintain momentum.^23^

#### 2. Political and Economic Factors

Political and economic factors have consequences for health outcomes and programming. Jamison et al. note there is a clear economic argument for investing in health. Reductions in mortality account for about 11% of recent economic growth in low- and middle-income countries, and a more comprehensive understanding of the economic value of investing in health provides a strong rationale for improved resource allocation across all sectors.^37^ However, economic downturns require governments to make difficult budgetary decisions between and within sectors. Changes in government and policies can also impact health priorities and outcomes. In Mexico, decentralization changed resource allocation needs during the transition plan, but the plan was not flexible enough to accommodate this change.^16^^,^^24^ Health reforms in Indonesia, which included decentralization, also affected the success of family planning graduation.^25^ Political and economic analyses should be undertaken during roadmap development to identify internal and external threats to the transition plan. In the Caribbean, some destabilizing factors include heavy debt burden and dependence on the tourism industry in a time of global austerity.

#### 3. Policy Environment

Policies and laws are important in outlining a country’s vision and communicating and regulating the role that local actors can play. Having appropriate policies in place to protect vulnerable populations, regulate the health sector, and provide guidance on the vision for HIV services are key indicators of the readiness of a country for transition. A critical lesson learned from family planning graduation programs is that policies are needed to protect the rights of individuals to access essential services.^16^ Cromer et al. note that national policies such as price controls, free distribution campaigns, and advertising restrictions create barriers for private-sector involvement in service delivery. It is also important to examine and strengthen the inclusiveness and data-driven character of the policymaking process.^28^

In the Caribbean, key policy areas to examine during transition are national strategic plans for health (inclusive of HIV), guidance and regulation for private-sector providers (for-profit and not-for-profit) of HIV services, and policies that protect the rights of vulnerable populations. Many Caribbean islands have allowed their strategic plans to lapse and have difficulty resourcing a thorough planning process. Many also lack policies to protect PLHIV and key populations.^39^ For example, sodomy laws are still enforced throughout much of the region, and adolescents often lack the right to access services without parental permission. These policies limit access to services among key populations and create barriers for providers working with these communities. Private providers and CSOs serving key populations will require ongoing external support to maintain gains made under PEPFAR until a more supportive policy environment and increases in domestic resources for these groups are available.^26^

Policies that protect the rights of vulnerable populations should be examined when transitioning HIV programs to country ownership.

#### 4. Alternative Funding Sources

Governments must identify ways to replace donor funding.^25^^,^^29^^,^^39^ This is difficult with even the strongest of government commitments because governments must balance competing priorities.^16^^,^^20^ Economic instability and increasing health care costs often drive health budgets below desired levels. The private health sector should be actively engaged to complement public services in a way that promotes efficient and cost-effective service delivery.^19^

HIV services in the Caribbean are often separate from other primary care services, which could perpetuate stigma.

Most Caribbean countries are undergoing health reforms to identify funding sources for soaring health care costs. Not all have determined what these reforms will look like nor have they conducted the research needed to make informed decisions. Several countries are considering national health insurance schemes and wish to design essential packages of services under those schemes. As a component of investment framework guidance from the Joint United Nations Programme on HIV/AIDS (UNAIDS), OECS countries have conducted quantitative analyses of trends in the HIV epidemic, the impact of prevention and treatment efforts to date, and a projection of possible future programming scenarios and their implications for the epidemic and program costs. The exercise produced strategic information to prioritize resources based on largest impact and spurred dialogue on identifying alternative funding sources in advance of transition.^40^ One identified alternative was leveraging private-sector resources, both human and financial. Examples include contracting private providers with specialty services and/or equipment not readily available in the public sector and formalizing arrangements with providers and local corporations to provide confidential, stigma-free counseling and testing services.

#### 5. Integration of HIV Programs

Bossert asserts that the sustainability of donor-funded programs relies on effective integration of programs into existing administrative structures.^39^ The evolution of PEPFAR and other large HIV donors has seen a shift away from siloed HIV programs to integrated service delivery models. Incorporating HIV into general Ministry of Health structures facilitates the integration of HIV services into primary care, resulting in improved management of and access to these services. In the Caribbean, most HIV programs have been integrated at least nominally within Ministry of Health structures. In practice, however, as evidenced by HIV-only clinic days in some countries, HIV services are often still separate from other primary care services in a manner that perpetuates stigma. Caribbean islands need assistance to fully integrate programs in a way that supports HIV services and protects the users of those services.

#### 6. Institutionalized Processes

To encourage sustainability, any process integral to a program needs to be institutionalized and standardized.^29^^,^^30^^,^^41^ Bradach notes that sustainable programs often require systems, structures, and processes to be standardized and articulated.^41^ Standardization may require the development of standard treatment protocols, guidelines for service delivery, clear job descriptions, checklists for service provisions and monitoring, standardized indicator sets, or other job-support tools. Standardizing and simplifying procedures also assist in overcoming human resource constraints.^29^ Bennett et al. are evaluating a PEPFAR-funded program in India where management shifted from NGOs to the government.^30^ Two key areas of evaluation are how well programs have been integrated into existing organizational systems and practices and the extent to which institutional standards guide program management. The evaluation framework assumes that institutionalization and standardization create processes to ensure program quality in activities that will be transitioned from the NGO to the government.

Health systems and private-sector assessments conducted in 6 OECS countries revealed that HIV programs are often more likely to have standardized procedures than programs in other health areas. This is especially true for M&E of antiretroviral therapy (ART) provision because it is driven by donor requirements and funding. Further support may be needed to develop and increase the use of standard treatment protocols and to regulate private facilities, including labs that provide testing for HIV. A Caribbean transition plan could provide further support to integrate and streamline the standards, guidelines, and M&E structures from HIV programs into the overall practice of the Ministries of Health.

#### 7. Procurement and Supply Chain Management

Pharmaceuticals and other commodities are an important part of ensuring access to HIV testing and treatment. This is an area in which international donors, especially the USG and the Global Fund, have made substantial investments. PEPFAR’s Track 1.0 ART program helped Ministries of Health in 13 countries strengthen supply chain management and antiretroviral (ARV) procurement. By 2011, the program had been successfully transitioned to country ownership and was providing ART for more than 925,000 patients.^42^ A key component of sustainable procurement and supply chain management involves building the capacity of in-country stakeholders to take responsibility for overseeing logistics and financing procurements.^16^^,^^20^^,^^25^ For example, commodities were largely funded and managed by USAID prior to graduation from family planning programs. Cromer et al. note that early graduates from USAID family planning funding had procurement systems in place but experienced stock-outs because they lacked experience procuring through different systems.^16^ PEPFAR’s capacity-building efforts in Botswana have increased access to essential drugs from 46% to 78% but have had difficulties addressing issues of poor storage and stock-outs.^20^

The USG has consistently supported procurement and supply chain management efforts in the Eastern Caribbean, including establishment of the OECS Pharmaceutical Procurement System (PPS). While research has shown that pooled procurement does not always result in the expected price drops due to market consolidation and the influence of a smaller number of producers, the OECS has experienced positive gains.^43^ Early results of the PPS have included increased bargaining power, average cost savings of 37% on selected purchases, enhanced quality control, and measurable increases in access to medicines.^44^ However, delayed payments have placed PPS under threat of collapse, which could lead to major challenges in procuring affordable ARVs when donor funds are no longer available. PPS has been working to improve forecasting and supply chain management, but continued capacity-building efforts are needed. Central Medical Stores in each country often face stock-outs of essential drugs, including ARVs, and testing reagents in part because there is low capacity for forecasting and monitoring of inventory. These challenges are intensified by a lack of cash flow within governments that prevent timely payments to manufacturers. Transition plans should consider building the capacity of PPS and local supply chain managers to improve and expand the current procurement system, including advocating to Ministries of Finance to ensure funding for essential medicines and commodities that are currently procured and supplied by donors but for which funding will be phased out in the near future. Adding these commodities to the pooled procurement system and ensuring timely payment could increase cost savings and improve efficiency similar to gains historically made under PPS.

#### 8. Staffing and Training Needs

The capacity and retention of skilled workers is essential to ensuring a smooth transition from donor support.^19^^–^^21^^,^^25^^,^^28^^,^^39^ While PEPFAR often supports seconded staff within Ministries of Health, decreases in funding mean that countries must intensify their hiring, retention, and training of health professionals to fill these gaps. For example, PEPFAR supported nearly 150 positions in Botswana, mainly in planning and strategic information.^20^ Decreases in PEPFAR funding meant that the government was faced with filling these positions alongside existing issues of major turnover and lack of key technical competencies in planning and management.^20^ Botswana was further constrained by macroeconomic policies that created hiring freezes at the urging of the International Monetary Fund (IMF).^20^

The Caribbean region has long suffered from brain drain, especially among nurses.^14^^,^^15^ Most Caribbean islands have health worker shortages, and public health management positions are difficult to fill with experienced personnel.^14^ Rotational patterns often mean that those who have been trained extensively in testing or other HIV services are rotated out of the facilities that host these services. Most countries in the region are currently working with partners to develop human resources for health strategies and train health workers. However, there will inevitably be a lag in the time needed to fully develop new and train existing cadres of workers. While there are currently few seconded positions in the Caribbean, strategic secondments during the transition process could help fill these gaps and assist in identifying areas for capacity building.

#### 9. Private Sector and Civil Society Engagement

In many countries, private-sector providers and NGOs have played a large role in delivering services, monitoring quality of public services, and/or advocating on behalf of marginalized groups. For example, NGOs and the for-profit private sector have played a large role in providing contraceptives. As a result, USAID focused its family planning graduation on developing sustainability of and business plans for private-sector providers to continue offering these services.^16^^,^^25^

In many countries, private providers and NGOs play a large role in delivering HIV services, especially to marginalized populations.

In cases where NGOs or private providers are delivering HIV services, transition will require them either to develop their own sustainability plans or to align with government norms to function under government auspices.^16^^,^^30^ In some countries, much work may need to be done to identify, establish, and/or formalize partnerships, networks, and roles between the government, private sector, and civil society.^28^ Investments in NGOs will help increase the sense of urgency and community engagement around HIV.^20^ When already an advocate, additional efforts should be made to ensure civil society has a place at the table for policymaking, especially to represent the needs of marginalized populations. In South Africa, for example, PEPFAR was a primary provider of services for key populations such as CSWs and MSM, often through support to NGOs. However, an initial assessment of transition there has shown that NGOs have suffered decreased implementation capacity and engagement as PEPFAR funding has shifted toward the public sector.^18^

The current and potential role of the private health sector to deliver HIV services in the Caribbean is vast. The NGO community performs a critical outreach function that provides prevention, counseling, testing, and other services to the populations most vulnerable to HIV. This service is often not feasible through public-sector facilities that are challenged by policies that criminalize the behaviors of key populations and prevent youth from accessing services without parental consent. Members of key populations may be reluctant to go to public-sector clinics for fear of limited confidentiality and the stigma associated with seeking services from the few facilities at which HIV is treated. Despite their critical role in national HIV responses, many Caribbean NGOs are either volunteer-based or heavily reliant on donor funding. For example, while the Caribbean HIV/AIDS Alliance (CHAA) was the leading provider of outreach services for key populations on most of the Eastern Caribbean islands, it was more than 95% reliant on PEPFAR funding.^45^ Critical NGOs will require support with developing and implementing funding diversification strategies and sustainability plans to ensure long-term viability and continued access to essential services for key populations.

Key population groups may be reluctant to seek HIV services from the public sector for fear of limited confidentiality and of stigma.

Private health care providers also possess a breadth and depth of experience in providing HIV care and treatment services throughout the region. Oftentimes overlooked, the private sector is poised to play a larger role in filling gaps in HIV programming. Recent mapping exercises of private-sector resources for health in 4 Caribbean countries conducted by the Strengthening Health Outcomes through the Private Sector (SHOPS) project found that the private health sector was much larger than originally understood, and many private providers had training in HIV counseling, testing, or treatment but were not using their skills due to lack of patient demand.^46^^–^^48^ These providers have the capacity to increase access to essential HIV services while providing the confidentiality that PLHIV seek. Activities geared toward greater private-sector engagement, ranging from fostering sustainable partnerships and policy dialogue to increasing access to training, will be crucial to sustaining health outcomes.

Private providers in the Eastern Caribbean are capable of playing a larger role in the HIV response.

## DISCUSSION

Our review of the literature has shown that transitions to country ownership, including some current PEPFAR transitions, face many barriers to success. Transitions have been hindered by donor-imposed timelines; the exclusion of key stakeholders in the planning process; lack of clear communication; and failure to ensure recipient countries have the resources and capacity to successfully maintain gains made under PEPFAR support. PEPFAR has been aiming to reverse this trend with a focus on country ownership in PEPFAR II and sustainability in PEPFAR 3.0. Our findings identified areas of overlap with the latest PEPFAR guidance and suggested additional points to consider for a successful transition.

Specifically, PEPFAR’s most recent COP Guidance has incorporated a new assessment of sustainability. As a part of the assessment, country programs are asked to “define gaps and bottlenecks, structural and cultural barriers.”^4^ The guidance suggests looking for gaps in resources, quality, data, efficiency, and structural and cultural barriers—key areas of health systems strengthening. These 5 areas align closely with our findings, which suggest the need to assess financial and human resources, commodity and supply chain management capacity, quality standards including the existence of guidelines for health workers, the use of data for decision making and program management, laws and policies, and engagement with the private sector and civil society. Based on our analysis, we also recommend assessing the commitment of country leadership in addressing the HIV response, potential threats in the political and economic environment, and integration of HIV programming into existing structures.

Our findings suggest that stakeholder engagement and clear communication are essential steps in a successful transition to country ownership. The new COP Guidance prioritizes stakeholder engagement and offers some insight on how to best engage private-sector stakeholders in program planning. Much of the guidance, however, places local stakeholders in a consultative role rather than prioritizing the primacy of the partner country in determining the way forward.^4^ PEPFAR itself has identified primacy of the partner country as a key part of sustainability planning elsewhere.^5^ This should be strongly reflected in all guidance documents to ensure sustained, meaningful involvement of country stakeholders. We would recommend that the operational planning process be undertaken with the involvement of national stakeholders from the beginning in roles beyond consultation. Our findings suggest that actively involving these stakeholders in the design and implementation of transition planning will be invaluable in strengthening plan design, ensuring country buy-in and ownership, and fostering a greater understanding of PEPFAR’s intentions.

PEPFAR transition guidance should clearly articulate the importance of meaningful involvement of local stakeholders in the transition process.

### Limitations

A major challenge in conducting this review was navigating the changing vernacular and perceptions of transition to country ownership among stakeholders. Oftentimes “graduation,” “transition,” “country ownership,” “sustainability planning,” and “donor withdrawal” were used interchangeably. The need for broad search terms produced a volume of results that made an exhaustive literature search unfeasible. In the future, a more systematic review could be beneficial particularly as more articles are produced regarding PEPFAR’s transition to greater country ownership. The review also relied on the existence of available literature that conformed to the defined research questions. Given the limited availability of scientifically conducted research on the topic area, the majority of available literature was project reports.

## CONCLUSION

Lessons learned from past and current transitions from donor-led programs to country ownership suggest that the first step in any successful transition to country ownership is mutually agreeing upon the goal and actions required and then developing and articulating a detailed yet flexible roadmap, in collaboration with relevant stakeholders to ensure buy-in and ownership of the process. Assessing readiness for such transition is challenging and must account for unique contextual factors across all facets of the health system. In the Caribbean, readiness for transition will require strengthening health systems, further engaging the private sector, and building the capacity of NGOs to take on essential program functions. Ongoing donor support for targeted capacity-building technical assistance and long-term M&E will be vital to ensuring that the countries of the Caribbean are able to take a leading role in their HIV responses while maintaining or improving upon the substantial gains made with PEPFAR support.

## Notes

**Peer Reviewed**

## References

[1] US President’s Emergency Plan for AIDS Relief (PEPFAR). World AIDS Day 2014 update: PEPFAR latest results fact sheet. Washington (DC): PEPFAR; [2014] Available from: http://www.pepfar.gov/funding/results/index.htm

[2] Office of the Global AIDS Coordinator (OGAC). PEPFAR blueprint: creating an AIDS-free generation. Washington (DC): OGAC; 2012 Available from: http://www.pepfar.gov/documents/organization/201386.pdf

[3] US President’s Emergency Plan for AIDS Relief (PEPFAR). PEPFAR 3.0. Controlling the epidemic: delivering on the promise of an AIDS-free generation. Washington (DC): PEPFAR; 2014 Available from: http://www.pepfar.gov/documents/organization/234744.pdf

[4] US President’s Emergency Plan for AIDS Relief (PEPFAR). PEPFAR country/regional operation plan (COP/ROP) 2015 guidance. Washington (DC): PEPFAR; 2015 Available from: http://www.pepfar.gov/documents/organization/237669.pdf

[5] US President’s Emergency Plan for AIDS Relief (PEPFAR). Sustainability planning document: advancing country ownership in PEPFAR III. Washington (DC): PEPFAR; 2013 Available from: http://www.pepfar.gov/documents/organization/217767.pdf

[6] Joint United Nations Programme on HIV/AIDS (UNAIDS). Regional factsheet 2012: Latin America and the Caribbean. Geneva: UNAIDS; [2012] Available from: http://www.unaids.org/en/media/unaids/contentassets/documents/epidemiology/2012/gr2012/2012_FS_regional_la_caribbean_en.pdf

[7] US President’s Emergency Plan for AIDS Relief (PEPFAR). Caribbean regional HIV and AIDS partnership framework 2010–2014. Washington (DC): PEPFAR; 2010 Available from: http://www.pepfar.gov/documents/organization/143196.pdf

[8] Stop AIDS Alliance. HIV and human rights in Latin America and the Caribbean. Hove (UK): International HIV/AIDS Alliance; 2010 Available from: http://www.aidsalliance.org/includes/document/PolicyBriefLACHumanRights.pdf

[9] Nakhimovsky S Brizan-St. Martin R Cogswell H Young D Theodore K St. Kitts and Nevis 2011 national health accounts and HIV subaccounts. Bethesda (MD): Abt Associates, Health Systems 20/20 Caribbean Project; 2013 Available from: https://www.hfgproject.org/saint-kitts-nevis-2011-national-health-accounts-hiv-subaccounts/

[10] Bhuwanee K Bethelmie D Cogswell H Young D Theodore K LaFoucade A Dominica 2010–2011 national health accounts and HIV subaccounts. Bethesda (MD): Abt Associates, Health Systems 20/20 Caribbean Project; 2013 Available from: https://www.hfgproject.org/dominica-2010-2011-national-health-accounts-hiv-subaccounts/

[11] PEPFAR Dashboards [Internet]. Washington (DC): US President’s Emergency Plan for AIDS Relief (PEPFAR). 2004 -. Caribbean region results, FY 2006–2013; [cited 2015 Feb 18] Available from: http://data.pepfar.net/country/impact?country = Caribbean%20Region&year = 2006&yearTo = 2013

[12] Healthy Caribbean Coalition. Chronic non communicable diseases (NCDs) in the Caribbean: the facts. Barbados: The Coalition; [200?]. Available from: http://www.healthycaribbean.org/UNHLM-HCC/Caribbean-NCDs-Fact-sheet.pdf

[13] Meyer PJ U.S. foreign assistance to Latin America and the Caribbean: recent trends and FY2015 appropriations. Washington (DC): Congressional Research Service; 2014 Available from: http://fas.org/sgp/crs/row/R43577.pdf

[14] Pan-American Health Organization (PAHO). Road map for strengthening the Caribbean health workforce, 2012–2017: draft [abridged version]. Washington (DC): PAHO; 2012 Available from: http://new.paho.org/hrhcaribbean/wp-content/uploads/2012/03/Roadmap-CaricomRH-2012.pdf

[15] Kurowski C Carpio C Vujicic M Gostin LO Baytor T Towards a regional strategy to strengthen the nurse workforce of the English-speaking CARICOM: international legal instruments, agreements and obligations. Washington (DC): International Bank for Reconstruction and Development/The World Bank; 2011 Available from: http://www-wds.worldbank.org/external/default/WDSContentServer/WDSP/IB/2012/06/07/000425962_20120607130200/Rendered/PDF/693740WP0Box360seWorkforce00PUBLIC0.pdf

[16] Cromer C Pandit T Robertson J Newijk A The family planning graduation experience: lessons for the future. Executive summary. Washington (DC): Population Technical Assistance Project; 2004 Available from: http://pdf.usaid.gov/pdf_docs/Pnadc236.pdf

[17] Brundage SC Terra nova: how to achieve a successful PEPFAR transition in South Africa. Washington (DC): Center for Strategic & International Studies; 2011 Available from: http://csis.org/files/publication/111205_Brundage_TerraNova_WEB.pdf

[18] Kavanagh MM The politics of transition & the economics of HIV/AIDS and PEPFAR in South Africa. Philadelphia (PA): University of Pennsylvania, Health Global Access Project; 2014 Available from: http://www.healthgap.org/_the_politics_of_transition_economics_of_hiv_aids_pepfar_in_south_africa

[19] US President’s Emergency Plan for AIDS Relief (PEPFAR). U.S. government interagency paper on country ownership. Washington (DC): PEPFAR; 2012 Available from: http://www.ghi.gov/principles/docs/ownershipInteragencyPaper.pdf

[20] Stash S Cooke J Fisher M Kramer A Competing pressures for U.S. PEPFAR in Botswana: rising ambitions, declining resources. Washington (DC): Center for Strategic & International Studies; 2012 Available from: http://csis.org/files/publication/121128_Stash_PEPFARBotswana_Web.pdf

[21] Slob A Jerve AM Managing aid exit and transformation: lessons from Botswana, Eritrea, India, Malawi and South Africa. Synthesis report. Stockholm: Swedish International Development Cooperation Agency (Sida);. 2008 Co-published by the Netherlands’ Ministry of Foreign Affairs, Danish International Development Agency (Danida) and the Norwegian Agency for Development Cooperation (Norad) Available from: http://www.cmi.no/publications/publication/?3155 = managing-aid-exit-and-transformation

[22] Hirschhorn LR Talbot JR Irwin AC May MA Dhavan N Shady R From scaling up to sustainability in HIV: potential lessons for moving forward. Global Health. 2013;9(1):57. 10.1186/1744-8603-9-57. 24199749 PMC3826849

[23] Franco L Miller L Browne C Mason G Sears C Midterm evaluation report: PEPFAR Caribbean regional program. Arlington (VA): USAID’s AIDS Support and Technical Assistance Resources, AIDSTAR-One, Task Order One; 2013 Available from: http://www.aidstar-one.com/sites/default/files/AIDSTAR-One_CaribbeanMidterm_Final_1.pdf

[24] Alkenbrack S Shepherd C Lessons learned from phaseout of donor support in a national family planning program: the case of Mexico. Washington (DC): Futures Group, Policy Project; 2005 Available from: http://www.policyproject.com/pubs/generalreport/Mexico_Phaseout_of_FP_Donor_Suppot_Report.pdf

[25] Betrand JT USAID graduation from family planning assistance: implications for Latin America. Washington (DC): Population Institute; 2011. Co-published by Tulane University School of Public Health and Tropical Medicine; 2011 Available from: http://www.populationinstitute.org/external/files/reports/FINAL_LAC_Report.pdf

[26] Collins C Beyrer C. Country ownership and the turning point for HIV/AIDS. Lancet Glob Health. 2013;1(6):e319–e320. 10.1016/S2214-109X(13)70092-5. 25104588

[27] Vermund SH Sidat M Weil LF Tique JA Moon TD Ciampa PJ. Transitioning HIV care and treatment programs in southern Africa to full local management. AIDS. 2012;26(10):1303–1310. 10.1097/QAD.0b013e3283552185. 22706012 PMC3576840

[28] Gutmann M Miller Franco L Capacity assessment tool for transitioning management and leadership of PEPFAR HIV care and treatment program to local partners: draft. Arlington (VA): USAID’s AIDS Support and Technical Assistance Resources, AIDSTAR-One, Task Order 1; 2011.

[29] Mangham LJ Hanson K. Scaling up in international health: what are the key issues? Health Policy Plan. 2010;25(2):85–96. 10.1093/heapol/czp066. 20071454

[30] Bennett S Singh S Ozawa S Tran N Kang J. Sustainability of donor programs: evaluating and informing the transition of a large HIV prevention program in India to local ownership. Glob Health Action. 2011;4. 10.3402/gha.v4i0.7360. 22184502 PMC3241943

[31] Sulzbach S Chankova S Tarantino L Feeley R Ingerson K Narcisse C Antigua and Barbuda health systems and private sector assessment 2011. Bethesda (MD): Abt Associates; 2012 Available from: https://www.hfgproject.org/antigua-barbuda-health-systems-private-sector-assessment-2011/

[32] Hatt L Altman D Chankova S Narcisse C Peña DL Riley P Grenada health systems and private sector assessment 2011. Bethesda (MD): Abt Associates; 2012 Available from: https://www.hfgproject.org/grenada-health-systems-private-sector-assessment-2011/

[33] Hatt L Vogus A O’Hanlon B Banke K Williamson T Hainsworth M Kitts and Nevis health systems and private sector assessment 2011. Bethesda (MD): Abt Associates; 2012 Available from: https://www.hfgproject.org/st-kitts-nevis-health-systems-private-sector-assessment-2011/

[34] Rodriguez M O’Hanlon B Vogus A Feeley R Narcisse C Saint Lucia health systems and private sector assessment 2011. Bethesda (MD): Abt Associates; 2012 Available from: https://www.hfgproject.org/saint-lucia-health-systems-private-sector-assessment-2011/

[35] Rodriguez M Williamson T Vogus A Macgregor-Skinner E Peña DL Wilson A Saint Vincent and the Grenadines health systems and private sector assessment. Bethesda (MD): Abt Associates; 2012 Available from: https://www.hfgproject.org/saint-vincent-grenadines-health-systems-private-sector-assessment/

[36] Sulzbach S Ingerson K Rodriguez M Williamson T Hainsworth M Fairbank A Dominica health systems and private sector assessment. Bethesda (MD): Abt Associates; 2012 Available from: https://www.hfgproject.org/dominica-health-systems-private-sector-assessment/

[37] Jamison DT Summers LH Alleyne G Arrow KJ Berkley S Bingawaho A Global health 2035: a world converging within a generation. Lancet. 2013;382(9908):1898–1955. 10.1016/S0140-6736(13)62105-4. 24309475

[38] Health Systems 20/20; SHOPS Project. Literature review on health systems and private sector in the Caribbean Region (draft). Bethesda (MD): Abt Associates; 2010.

[39] Bossert TJ. Can they get along without us? Sustainability of donor-supported health projects in Central America and Africa. Soc Sci Med. 1990;30(9):1015–1023. 10.1016/0277-9536(90)90148-L. 2336568

[40] Hamilton M Musau S Sustaining the HIV and AIDS response in countries of the OECS: regional investment case analysis. Bethesda (MD): Abt Associates; 2014.

[41] Bradach J Going to scale: the challenge of replicating social programs. Stanf Soc Innov Rev. Standford (CA): Stanford Graduate School of Business; Spring 2003 Available from: http://www.ssireview.org/articles/entry/going_to_scale

[42] El-Sadr WM Holmes CB Mugyenyi P Thirumurthy H Ellerbrock T Ferris R Scale-up of HIV treatment through PEPFAR: a historic public health achievement. J Acquir Immune Defic Syndr. 2012;60 (Suppl 3):S96–S104. 10.1097/QAI.0b013e31825eb27b. 22797746 PMC3445041

[43] Waning B Kyle M Diedrichsen E Soucy L Hochstadt J Bärnighausen T Intervening in global markets to improve access to HIV/AIDS treatment: an analysis of international policies and the dynamics of global antiretroviral medicines markets. Global Health. 2010;6:9. 10.1186/1744-8603-6-9. 20500827 PMC2883977

[44] World Health Organization (WHO). Multi-country regional pooled procurement of medicines. Identifying key principles for enabling regional pooled procurement and a framework for inter-regional collaboration in the African, Caribbean, and Pacific Island Countries. Geneva: WHO; 2007 Available from: http://apps.who.int/medicinedocs/documents/s14862e/s14862e.pdf

[45] Tarantino L Ingerson K Chadbourn C Financial sustainability recommendations for the Caribbean HIV/AIDS Alliance (CHAA): draft report. Bethesda (MD): Abt Associates; 2014.

[46] Ingerson K Private sector resources for health in Antigua and Barbuda: a rapid mapping. Bethesda (MD): Abt Associates; 2012.

[47] Tuchman J Private sector resources for health in Dominica: a rapid mapping. Bethesda (MD): Abt Associates; 2012.

[48] Ingerson K Private sector resources for health in St. Kitts and Nevis: a rapid mapping. Bethesda (MD): Abt Associates; 2012.

